# Investigation of Acinetobacter baumannii Outbreak in a Burn Unit Revealed a Surprising Environmental Source

**DOI:** 10.7759/cureus.42984

**Published:** 2023-08-05

**Authors:** Pradeep Kumar Mada, Mohammed J Alam

**Affiliations:** 1 Infectious Diseases, Comanche County Memorial Hospital, Lawton, USA; 2 Infectious Diseases, Louisiana State University Health Sciences Center, Shreveport, USA

**Keywords:** table fan, hospital environment, outbreak management, burn unit, acinetobacter baumannii

## Abstract

We describe an outbreak of *Acinetobacter baumannii* in a 15-bed regional burn unit in an academic tertiary care medical center, and the investigations and control measures used to halt the outbreak are described. Nine cases of *A. baumannii *were reported in our burn unit in a one-year period, which was higher than our expected number of two-three cases per year. Our burn unit director requested an outbreak investigation, and our hospital’s infection control department investigated thoroughly and found a source for that outbreak, which was never reported as a source in the previous literature. We identified table fans as the source of this outbreak. We then developed a strict fan policy, and after implementation of the policy, and terminal cleaning of rooms, only two cases per year of *A. baumannii *were reported in the next three-year period. We concluded that the table fans were colonized with *A. baumannii *and since they were used interchangeably in all patient rooms, caused this outbreak. There are no specific joint commission guidelines for the use of fans in hospitals. While fans can be used for the comfort of the patient, we should be cautious not to spread infections.

## Introduction

*Acinetobacter baumannii* is a gram-negative, aerobic, pleomorphic bacillus that is most commonly associated with hospital infections [1]. Outbreaks of *A. baumannii* infections in hospitals have been reported throughout the world, most commonly in augmented care units [2]. *Acinetobacter *spp., other than hospital-associated infections, is frequently associated with aquatic environments and soil. The organism has been found to colonize the skin and the respiratory and oropharyngeal secretions of individuals infected with it [3].

*A. baumannii* is implicated in the causation of diseases, ranging from pneumonia to serious blood or wound infections. *Acinetobacter *spp. has also been reported to colonize asymptomatically in tracheostomy sites or open wounds. Epidemiological trends of infections caused by *A. baumannii* reveal the emergence of multidrug-resistant strains over the last two decades [4]. The synergistic effect of this emerging antibiotic resistance and the ability of the organism to survive in hospital environments for more extended periods of time have contributed to the increased incidence of outbreaks caused by the organism. *A. baumannii* is associated with 80% of the reported infection outbreaks caused by *Acinetobacter *spp. in healthcare settings [5].

## Materials and methods

In this manuscript, we describe an outbreak of *A. baumannii* in our burn unit, which occurred from January 2009 to November 2009. Nine cases occurred in a 15-bed regional burn unit of an academic tertiary care medical center in one year period, which was higher than our expected number of two-three cases per year. A detailed epidemiological investigation of the outbreak was performed to identify the source of infection. The control measures that were initiated to terminate the spread of infection and the outcomes of these measures will be described.

Burn unit setup

Our burn unit has a total of 15 beds, of that four intensive care unit (ICU) private rooms, nine beds on the convalescent end (five single rooms, two double occupancy), and two overflow beds are there. The unit also has one burn operating room (OR). As we mentioned, all ICU rooms are private, and the convalescent end has a combination of private and shared. However, when patients are placed in isolation, they would be in a private room. The majority of the ICU, hydrotherapy, and OR areas are supplied by two air-handling units located on the rooftop. These units recirculate the air through 95% efficiency (minimum efficiency reporting values (MERV) 14) air filters while pretreating the required outdoor airflow into the ICU. The remaining patient rooms and nurse stations are served by individual fan coil units that recirculate the room air through cooling/heating coils and 25% efficient (MERV 7) air filters. These individual rooms also have the required amount of outside air ducted into the rooms that were pretreated through MERV 14 filtration. Any isolation room present has 100% of the air exhausted out of the building and is under negative pressure. The standard ICU and patient rooms should be neutral with the single OR under positive pressure.

Infection outbreak and control

A total of nine patients have documented infections caused by *A. baumannii, *and in those, three cases were hospital-acquired pneumonia, four cases were skin and soft tissue infections, and two cases were urinary tract infections (Table 1). For this study, a case of *A. baumannii *infection is defined as clinically apparent infection due to the organism after 48 hours of stay in the burn unit. The following methods were used to establish the diagnosis of *A. baumannii.*

**Table 1:** Acinetobacter baumannii infection case list TBSA: total body surface area; LTAC: long-term acute care hospital; UTI: urinary tract infection

Patient no	Diagnosis	Admission date	Acinetobacter baumanniiinfection diagnosis date	Risk factors	Outcome
1	Pneumonia	12/25/08	1/9/09	Intubation/ >20% TBSA	Discharged to LTAC
2	Pneumonia	9/17/08	2/13/09	Intubation/ >10% TBSA	Discharged to LTAC
3	Pneumonia	9/2/08	3/20/09	Intubation/ >20% TBSA	Discharged to LTAC
4	Skin and soft tissue infection	10/7/08	11/3/09	>20% TBSA	Deceased
5	Skin and soft tissue infection	2/3/09	6/21/09	>20% TBSA	Discharged to LTAC
6	Skin and soft tissue infection	4/21/09	5/06/09	>10% TBSA	Discharged to LTAC
7	Skin and soft tissue infection	5/8/09	10/09/09	>20% TBSA	Deceased
8	UTI	5/11/09	6/30/09	Foley catheter, >10% TBSA	Discharged to LTAC
9	UTI	6/23/09	11/25/09	Foley catheter, >20% TBSA	Discharged to LTAC

Urine culture

Urine cultures were plated using a calibrated loop to appropriate media to recover Gram-negative and Gram-positive organisms. Cultures were held for a minimum of 48 hours before being discarded as “No Growth.” All growth was reported using colony counts. Organisms were identified using the Microscan instrument (Omron Microscan Systems, Inc., Washington, USA), and minimum inhibitory concentrations (MIC) susceptibility testing was conducted.

Bronchoalveolar lavage (BAL) bulture

Bronchoalveolar lavage cultures were plated to appropriate media using the 1 µL and 10 µL loops. Cultures were held for three days before discarding as "No Growth." All growth was reported using colony counts. Pathogens were reported with identification and susceptibility MIC results if the colony count was more than 1000 colony-forming units (CFU)/ml. Other isolates were only identified in the final report.

The antibiotic sensitivity of the infecting organism has been tested against antibiotics, including ampicillin/sulbactam, ceftazidime, meropenem, amikacin, gentamicin, tobramycin, levofloxacin, minocycline, colistin, and trimethoprim/sulfamethoxazole. The susceptibility of antibiotics was similar in all of the cases that were reported in the patients admitted to the burn unit. *A. baumannii* was resistant to ceftazidime, meropenem, amikacin, gentamicin, tobramycin, levofloxacin, trimethoprim/sulfamethoxazole, minocycline, and sensitive to colistin.

The frequent occurrence of the *A. baumannii* infection led to the notification of the Infection Control Team, which started an investigation into the outbreak in December 2009. Initial control measures include discarding all supplies in the hydrotherapy room, and that room was terminally cleaned every morning, and after every patient’s therapy was completed. We looked into hand hygiene audits for January to November 2009, which was reported as 97%, and hand hygiene/blood-borne pathogen supervisors' monthly checklist compliance was 100%. Environmental cultures were done at the request of the Infection Control Team. Swabs are collected from various surfaces, including hydro beds, hydro carts, hydro drain pans, hydro nozzles, hydro faucets, patient beds, sinks, bed tables, beds, burn unit lights, anesthesia carts, supply carts, scrub sinks, anteroom sinks, patient room faucets, and ventilators. Swabs were plated out to standard media and held for five days before being discarded as “No Growth in five days.” Bacteria isolates were identified to the genus and species levels.

## Results

The samples initially tested for the presence of *A. baumannii* were negative, including unit sinks, hydrotherapy beds, operating room tables, sinks, patient room door handles, and air conditioning grills. Later, two fans in the burn unit that were used on an intermittent basis in the burn unit were sampled, and they showed the presence of multi-drug resistance (MDR) *A. baumannii *(Table 2). The antibiotic sensitivity of the bacterium tested from the fans was similar to the sensitivity spectrum of the bacterium isolated from the patients reported from the burn unit. This incident led to the conclusion that these fans were the source of the outbreak since no other environmental cultures grew *A. baumannii.*

**Table 2:** Environmental source swab microbiology report

Environmental source sample	Date of collection	Culture report
Unit sink 1	12/20/09	Citrobacter freundii, Pseudomonas fluorescens putida
Unit sink 2	12/20/09	Citrobacter freundii
Unit sink 3	12/20/09	Fusarium, Pseudomonas aeruginosa
Hydro room; hydro bed & handles, glove boxes	12/30/09	No growth
OR supplies/supply cart	12/30/09	No growth
OR table, sink	12/30/09	No growth
Unit supply carts	12/30/09	No growth
Computers at the nursing station	01/15/10	Staphylococcus epidermidis
Patient rooms door handles, drawer handles	01/15/10	Staphylococcus aureus
Two fans in the unit	01/30/2010	Acinetobacter baumannii
Air conditioning grilles	01/25/2010	Staphylococcus aureus

Intervention

Once the source was identified, these fans were removed from the burn unit and were replaced with newer ones. We also performed terminal cleaning of the entire unit, including nurse stations, OR, and hydrotherapy rooms. Undiluted bleach was poured down into sinks and hydro drains to flood the drains so that no *Acinetobacter *spp. would survive, if it made its way to the hydrotherapy treatment bed. We used various disinfectants, such as QT-TB (n-alkyl (60% C14, 30% C16, 5% C12, 5% C18) dimethyl benzyl ammonium chlorides of 0.105%, n-alkyl (68% C12, 32% C14) dimethyl ethyl benzyl ammonium chlorides of 0.105%), Virex II 256 (didecyl dimethyl ammonium chloride), and Viracept (hydrogen peroxide of 3.130%; octanoic acid of 0.099%; peroxyacetic acid of 0.050%) applied as per manufacture recommendations and dwell time. We also continued cohorting MDR *A. baumannii-*colonized or *A. baumannii*-infected patients and cohorting staff related to MDR *A. baumannii *by assigning personnel to care only for patients known to be colonized or infected with MDR *A. baumannii.*

**Figure 1:**
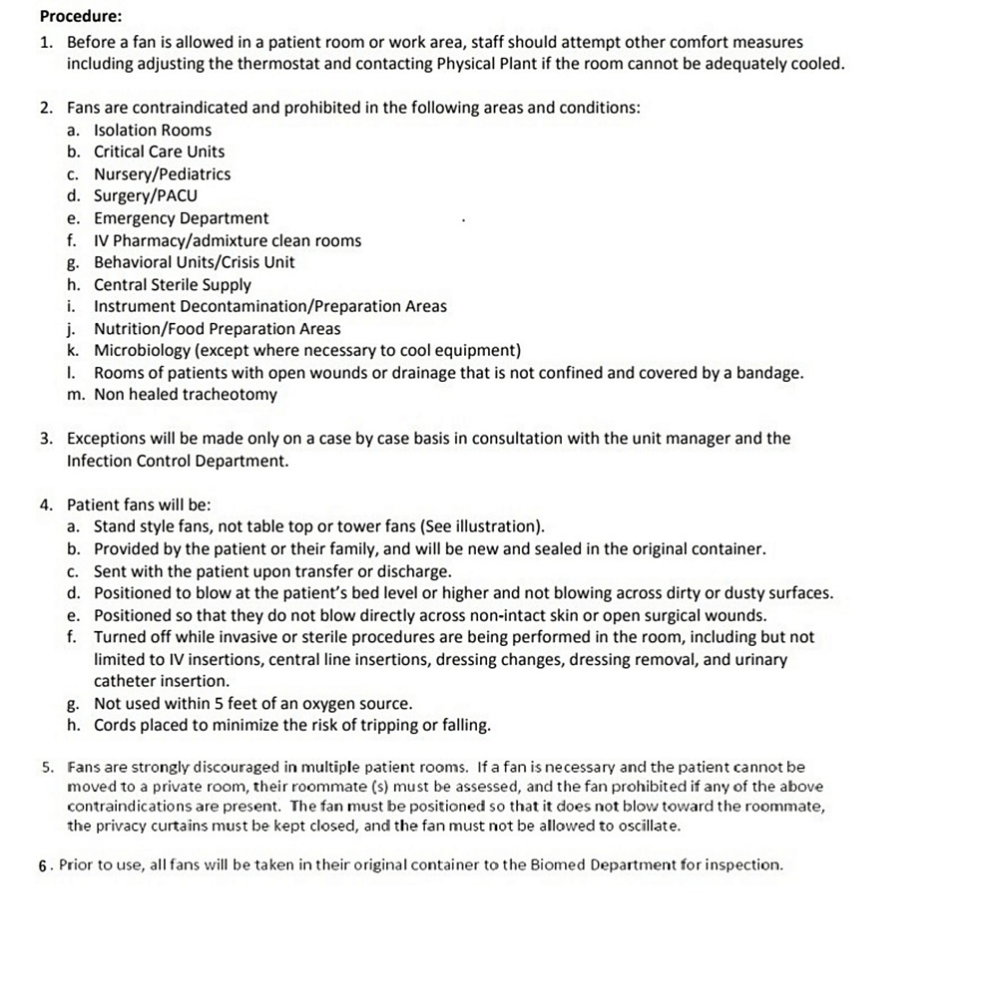
Fan policy in the burn unit

A strict fan policy was implemented (Figure 1). Along with the replacement of the identified sources of infection, measures such as distributing flyers for awareness on hand hygiene, ensuring dedicated trained staff for the burn unit, ensuring nightly terminal cleaning of the burn room using foot pedal laundry cart throughout the unit, replacing the cloth supply cart covers with vinyl, and establishing a house-wide protocol for the beds to be cleaned prior to patients entering the room were undertaken.

The effectiveness of intervention

Before the replacement of the fans, nine cases were reported over a one-year period. After the fans that were identified as the source of the infection were removed and replaced with new fans, along with other infection control measures, only two cases of *A. baumannii* per year were reported over the next three years.

## Discussion

*A. baumannii* is an opportunistic pathogen that is commonly associated with hospital-acquired infections in the United States. The organism is frequently related to aquatic environments, but it also can colonize the respiratory and oropharyngeal secretions of susceptible individuals [6]. The World Health Organization has grouped *Enterococcus faecium, Staphylococcus aureus, Klebsiella pneumoniae, A. baumannii, Pseudomonas aeruginosa*,* and Enterobacter *spp. (ESKAPE) as the most common and severe multidrug-resistant pathogens [7]. 

The patients are often uncomfortable and find the use of a fan soothing. The burn patients being heavily colonized and disseminating vast numbers of contaminated skin scales with* A. baumannii*, the ability of surviving better than most Gram-negative organisms in dry conditions, and free-standing fans move air and skin scales contaminated with *A. baumannii *greater than would be by normally air-conditioned rooms disseminating directly onto the patient’s body caused an outbreak in our burn unit.

An outbreak of *A. baumannii* can occur in a burn unit due to staffing shortages and lapses in compliance with infection prevention and control measures, resulting in an increased risk of cross-transmission between patients [8]. The outbreak in this instance appears to be initiated by the fans that were used in the burn unit. The fans were confirmed as the source of the infection because the antibiotic sensitivity pattern was similar to the isolates cultured from the patients infected with the organism. Fans are commonly used in burn units in hospitals, but no previous studies have reported them as a source of *A. baumannii *infection. The findings of the study underline the importance of environmental disinfection in managing the outbreak of *A. baumannii* infection, as reported in previous studies [9, 10]. Previous studies have reported that complete closure of units was essential to control outbreaks, but that was not required in this case [11]. However, environmental disinfection alone might not be sufficient in every case of infection outbreak by multidrug-resistant *A. baumannii.* Other concomitant infection control measures include isolation of patients, contact precautions, proper disposal of soiled linen, and education of visitors about outbreaks with the organism [12, 13].

There have been several reports of *A. baumannii *outbreaks in the past several years. The incidence of *A. baumannii *has led to precaution and action guidelines, such as the Association for Professionals in Infection Control and Epidemiology (APIC) Guide, where they cover the most common environmental shelters for *Acinetobacter *spp.: curtains, mattresses and pillows, wipes and cloth, dust, toys, and water pipes [14].

Effective measures to control a multidrug-resistant *A. baumannii* outbreak include contact precautions, proper hand hygiene, personal protective equipment, identifying source, patient isolation, terminal clean of the whole unit, and awareness raising among staff along with other policy and equipment changes. On top of this, a patient fan use guideline was created, which included measurements such as absolute requirements for fan usage, specific contraindications, instructions that must be done in each patient with a fan, and a complete revision of all fans prior its usage. There is no previously documented evidence/published data that table fans spreading infections in ICUs or burn units. Most hospitals do not have specific guidelines on the use of portable fans in hospital areas especially in private patient rooms, such as ICUs and burn units. However, it is prudent to have a policy where the Infection Control Team evaluates the infection risk associated with usage of fans. Our article highlights that there is a potential that fans can spread and cause subsequent infections, so we need to make sure of cleanliness of fans before each use. Patient comfort and overall patient care should be balanced with a strict fan policy. The key components to prevent transmission of *A. baumannii* in healthcare setting are periodic risk assessments, microbial surveillance, antibiotic stewardship, hand hygiene, standard and contact precautions, disinfection of surfaces, and disinfection of mobile medical equipment.

Limitations of the study

This is a retrospective descriptive study. Since there was no electronic medical record at that time, MIC details of strains are not available at this time. Only symptomatic patients were tested/identified, and the patients admitted to this unit were routinely not screened for colonization with *Acinetobacter* on admission. Hospital staff was also not tested for carriage. The outbreak in our burn unit was controlled after executing of numerous measures, and it is difficult to pinpoint which measure eventually controlled the outbreak.

## Conclusions

This article underlines the importance of an outbreak investigation and environmental sampling in implementing the appropriate infection control strategies for an outbreak. Effective measures to control a multidrug-resistant *A. baumannii *outbreak include contact precautions, proper hand hygiene, personal protective equipment, identifying sources, patient isolation, terminal cleaning of the whole unit, awareness raising among staff, and other policy and equipment changes. Most hospitals do not have specific guidelines on the use of portable fans in hospital areas, especially in private patient rooms, such as ICUs and burn units. However, it is prudent to have a policy where the Infection Control Team evaluates the infection risk associated with the usage of fans. Our article highlights that there is a potential that fans can spread and cause subsequent infections.
